# Molecular-phylogenetic analyses of *Ixodes* species from South Africa suggest an African origin of bird-associated exophilic ticks (subgenus *Trichotoixodes*)

**DOI:** 10.1186/s13071-023-05998-5

**Published:** 2023-10-28

**Authors:** Sándor Hornok, Jenő Kontschán, Nóra Takács, Heloise Heyne, Áron Botond Kovács, Olivier Plantard, Gergő Keve, Denis Fedorov, Miklós Gyuranecz, Ali Halajian

**Affiliations:** 1 Department of Parasitology and Zoology, University of Veterinary Medicine, Budapest, Hungary; 2 HUN-REN-UVMB Climate Change: New Blood-Sucking Parasites and Vector-Borne Pathogens Research Group, Budapest, Hungary; 3 Plant Protection Institute, HUN-REN Centre for Agricultural Research, Budapest, Hungary; 4 Department of Plant Sciences, Albert Kázmér Faculty of Mosonmagyaróvár, Széchenyi István University, Mosonmagyaróvár, Hungary; 5 Epidemiology, Parasites & Vectors (EPV), ARC-Onderstepoort Veterinary Research (ARC-OVR), Onderstepoort, South Africa; 6 HUN-REN Veterinary Medical Research Institute, Budapest, Hungary; 7 National Laboratory of Infectious Animal Diseases, Antimicrobial Resistance, Veterinary Public Health and Food Chain Safety, HUN-REN Veterinary Medical Research Institute, Budapest, Hungary; 8 Oniris, INRAE, BIOEPAR, Nantes, France; 9 National Laboratory of Health Safety, HUN-REN Veterinary Medical Research Institute, Budapest, Hungary; 10 Research Administration and Development, University of Limpopo, Sovenga, 0727 South Africa; 11 Department of Biodiversity, DSI-NRF SARChI Chair, University of Limpopo, Sovenga, 0727 South Africa

**Keywords:** *Cox*1 gene, 16S rRNA gene, Carnivora, Eulipotyphla, Passeriformes

## Abstract

**Background:**

Among hard ticks (Acari: Ixodidae), the genus *Ixodes* comprises the highest number of species, which in turn are most numerous in the Afrotropical zoogeographic region. In South Africa extensive morphological studies have been performed on *Ixodes* species but only few reports included molecular analyses.

**Methods:**

In this study, 58 *Ixodes* spp. ticks, collected from ten mammalian and eight avian host species in South Africa, were molecularly and phylogenetically analyzed. In addition, a newly collected sample of the Palearctic *Ixodes trianguliceps* was included in the analyses.

**Results:**

Among the ticks from South Africa, 11 species were identified morphologically. The majority of ticks from mammals represented the *Ixodes pilosus* group with two species (*n* = 20), followed by ticks resembling *Ixodes rubicundus* (*n* = 18) and *Ixodes alluaudi* (*n* = 3). In addition, single specimens of *Ixodes rhabdomysae*, *Ixodes ugandanus*, *Ixodes nairobiensis* and *Ixodes simplex* were also found. Considering bird-infesting ticks, *Ixodes theilerae* (*n* = 7), *Ixodes uriae* (*n* = 4) and ticks most similar to *Ixodes daveyi* (provisionally named *I.* cf. *daveyi*, *n* = 2) were identified. Molecular analyses confirmed two species in the *I. pilosus* group and a new species (*I.* cf. *rubicundus*) closely related to *I. rubicundus *sensu stricto. Phylogenetic trees based on concatenated mitochondrial or mitochondrial and nuclear gene sequences indicated that the subgenus *Afrixodes* forms a monophyletic clade with bird-associated exophilic ticks (subgenus *Trichotoixodes*). *Ixodes trianguliceps* clustered separately whereas *I. alluaudi* with their morphologically assigned subgenus, *Exopalpiger*.

**Conclusions:**

Phylogenetic analyses shed new lights on the relationships of *Ixodes* subgenera when including multiple sequences from subgenus *Afrixodes* and African as well as Palearctic species of subgenera *Trichotoixodes* and *Exopalpiger*. Subgenera *Afrixodes* and bird-associated *Trichotoixodes* share common ancestry, suggesting that the latter might have also originated in Africa. Regarding the subgenus *Exopalpiger*, *I. alluaudi* is properly assigned as it clusters among different Australian *Ixodes*, whereas *I. trianguliceps* should be excluded.

**Graphical Abstract:**

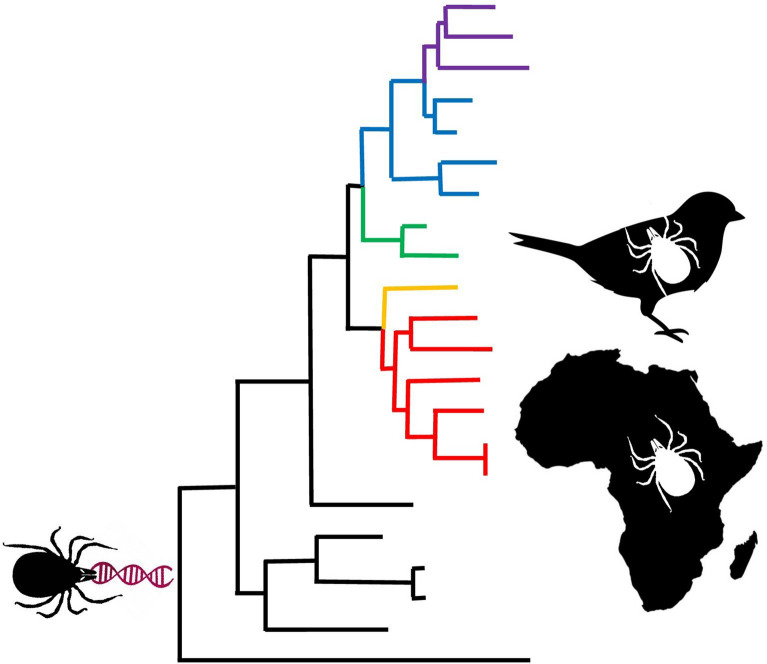

**Supplementary Information:**

The online version contains supplementary material available at 10.1186/s13071-023-05998-5.

## Background

Hard ticks (Acari: Ixodidae) and pathogens transmitted by them affect animal and human health worldwide, causing significant economic losses [1]. Currently, there are more than 760 species of ixodid ticks known to science [2], but this number is steadily increasing as the description of new species continues. The great majority of hard tick species belong to the genus *Ixodes* Latreille, 1795, currently consisting of 266 species [2]. Based on morphological considerations, this genus was divided into 14 subgenera [3] but later several new subgenera were proposed [4]. However, molecular analyses challenged the existence of some of those *Ixodes* subgenera [5, 6].

It is the Afrotropical zoogeographic region where the highest number of *Ixodes* species (i.e., 71 species) are indigenous [2]. The majority occurring in sub-Saharan Africa belong to the subgenus *Afrixodes* Morel, 1966. This is probably the most species-rich subgenus in *Ixodes* [3], comprising at least 60 species [7]. These are almost exclusively distributed in the Afrotropical zoogeographic region, including six species from Madagascar, but two species are present only in the Oriental zoogeographic region [3]. Females of this subgenus share the following morphological characters: long and narrow palps, well-developed auriculae, coxa I mostly with internal spur, the presence of syncoxae on coxae I–III and a circular anal groove [3]. However, for a significant number of *Afrixodes* species not all developmental stages are known or only the male or the female was described [8]. The number of *Afrixodes* species will probably increase, because from time to time new species are described [9], and others have been long known to exist but were not yet separately named and established, most notably in the so-called *Ixodes pilosus* group [10, 11]. The above data highlight the taxonomic importance and need for studying African *Ixodes* species.

The number of *Ixodes* species occurring in South Africa is at least 23 [2], with a few additional species only occasionally found [11, 12]. In this country extensive morphological studies have been performed on *Ixodes* species [11, 13–16] but only few reports included molecular analyses [7]. In addition, although large-scale molecular-phylogenetic studies focusing on ticks in general have been published, they included only one or two *Afrixodes* species [17–19].

Thus, the present study was initiated to compensate for this relative scarcity of molecular data on African *Ixodes* species, providing up to four (two mitochondrial, two nuclear) genetic markers (the cytochrome *c* oxidase subunit I [*cox*1] and 16S rRNA, as well as the 18S and 28S rRNA genes, respectively) for their phylogenetic analyses. Molecular-phylogenetic data reported herein are also meant as an initiative towards the barcoding of *Ixodes* species in South Africa, entailing clarification of their taxonomic status.

## Methods

### Sample collection and morphological analyses

*Ixodes* spp. ticks were collected from ten mammalian and eight avian species between May 2016 and November 2019 in South Africa. Data of location, season and host species are summarized in Table 1. All ticks were stored in 96% ethanol. Tick species were morphologically identified according to standard keys and illustrations [8, 11, 20–23]. Pictures were made with a VHX-5000 digital microscope (Keyence Co., Osaka, Japan).

**Table 1:** Data of collection (host, location, season), species and molecular data of ticks examined in this study

Host class	Host order	Host species	Location (season)	Tick species (*Ixodes*)*	GenBank accession numbers: long (or short) sequence			
					cox1	16S	18S	28S
Mammalia	Carnivora	*Canis mesomelas*	Limpopo Province	*I.* cf.* rubicundus*	OQ921970	OQ942715	OQ924742	OQ924936
		*Canis lupus familiaris*	Polokwane, Limpopo Province (winter)	*I. pilosus* group sp. I	OQ921940	OQ942680	OQ924736	OQ924930
				*I. pilosus* group sp. I	OQ921941	OQ942681	–	–
				*I. pilosus* group sp. I	OQ921942	OQ942682	–	–
			Kurisa Moyo, Limpopo Province (spring)	*I. pilosus* group sp. I	OQ921943	OQ942683	–	–
				*I. pilosus* group sp. I	–	OQ942684	–	–
				*I. pilosus* gr. sp. I. (M)	OQ921944	OQ942685	–	–
				*I. pilosus* group sp. I	OQ921945	OQ942686	–	–
				*I. pilosus* group sp. I	OQ921946	OQ942687	–	–
				*I. pilosus* group sp. I	OQ921947	OQ942688	–	–
			Haenertsburg, Limpopo Province (winter)	*I. pilosus* group sp. I	OQ921948	OQ942689	–	–
		*Civettictis civetta*	Haenertsburg, Limpopo Province (autumn)	*I. pilosus* group sp. II	OQ921974	OQ942721	OQ924746	OQ924946
				*I. pilosus* group sp. I	–	OQ942722	–	–
				*I. pilosus* group sp. I	OQ921975	OQ942723	–	–
				*I. pilosus* group sp. I	OQ921976	OQ942724	–	–
				*I. pilosus* group sp. I	OQ921977	OQ942725	(OQ924747)	(OQ924947)
				*I. pilosus* group sp. I	OQ921978	OQ942726	–	–
				*I. pilosus* group sp. II	–	OQ942727	–	–
				*I. pilosus* group sp. II	–	OQ942728	–	–
				*I. pilosus* group sp. I	OQ921979	OQ942729	–	–
				*I. pilosus* group sp. I	OQ921981	OQ942731	(OQ924747)	(OQ924947)
				*I. ugandanus*	OQ921980	OQ942730	OQ924748	OQ924948
			Polokwane, Limpopo Province (summer)	*I.* cf. *rubicundus* (M)	OQ921949	OQ942690	OQ924737	OQ924931
			Mokopane, Limpopo Province (spring)	*I.* cf. *rubicundus* (M)	OQ921950	OQ942691	OQ924737	OQ924931
				*I.* cf.* rubicundus*	OQ921951	OQ942692	–	–
				*I.* cf.* rubicundus*	OQ921952	OQ942693	–	–
				*I.* cf.* rubicundus*	OQ921953	OQ942694	–	–
				*I.* cf.* rubicundus*	OQ921954	OQ942695	–	–
				*I.* cf.* rubicundus*	OQ921955	OQ942696	–	–
				*I.* cf.* rubicundus*	OQ921956	OQ942697	–	–
				*I.* cf.* rubicundus*	OQ921957	OQ942698	–	–
				*I.* cf.* rubicundus*	OQ921958	OQ942699	–	–
		*Genetta* sp.	Limpopo Province	*I.* cf.* rubicundus*	OQ921971	OQ942716	OQ924742	OQ924936
				*I.* cf.* rubicundus*	OQ921972	OQ942717	–	(OQ924942)
		*Ichneumia albicauda*	Makhado (Louis Trichardt) (summer)	*I.* cf.* rubicundus*	OQ921963	OQ942707	OQ924742	OQ924936
		*Atilax paludinosus*	Mokopane, Limpopo Province (summer)	*I. nairobiensis*	–	OQ942705	OQ924741	OQ924935
	Eulipotyphla	*Atelerix frontalis*	Polokwane, Limpopo Province (summer)	*I.* cf.* rubicundus*	–	OQ942700	(OQ924738)	–
				*I.* cf.* rubicundus*	OQ921959	OQ942701	(OQ924739)	–
				*I.* cf.* rubicundus*	OQ921960	OQ942702	–	–
				*I.* cf.* rubicundus*	OQ921962	OQ942706	OQ924742	OQ924936
		*Crocidura silacea*	Makhado (Louis Trichardt) (autumn)	*I. alluaudi* (L)	–	–	–	–
				*I. alluaudi* (N)	–	–	–	(OQ924933)
				*I. alluaudi* (N)	–	OQ942704	–	(OQ924934)
	Chiroptera	*Miniopterus natalensis*	Venterskroon, North West Province (spring)	*I. simplex* (N)	–	OQ942713	–	OQ924939
	Primates	*Otolemur crassicaudatus*	Makhado (Louis Trichardt) (autumn)	*I. rhabdomysae*	OQ921961	OQ942703	OQ924740	OQ924932
Aves	Sphenisciformes	*Eudyptes chrysocome*	Marion Island (spring)	*I. uriae*	OQ921965	OQ942709	–	–
				*I. uriae*	OQ921966	OQ942710	OQ924744	OQ924938
				*I. uriae*	OQ921967	OQ942711	–	–
				*I. uriae*	OQ921968	OQ942712	–	–
	Passeriformes	*Euplectes orix*	Leeupan, North West Province (spring)	*I. theilerae*	OQ921964	OQ942708	(OQ924743)	OQ924937
		*Euplectes afer*	Limpopo Province	*I. theilerae*	–	–	–	–
		*Quelea quelea*	Limpopo Province	*I. theilerae*	OQ921969	OQ942714	(OQ924745)	OQ924940
		*Ploceus capensis*	Worcester, Western Cape Province	*I. theilerae*	–	-	–	(OQ924941)
		*Ploceus velatus*	Hoopstad, Free State Province (spring)	*I. theilerae*	–	–	–	–
				*I. theilerae*	–	OQ942719	–	(OQ924944)
				*I. theilerae*	–	–	–	–
		*Cossypha caffra*	Limpopo Province (summer)	*I.* cf.* daveyi*	–	OQ942718	–	(OQ924943)
		*Cossypha dichroa*	Mashishing (Lydenburg), Mpumalanga Province (summer)	*I.* cf.* daveyi*	OQ921973	OQ942720	–	(OQ924945)

^*^Examined tick specimens were females unless otherwise indicated (M male, N nymph, L larva)

### DNA extraction and PCR analyses

Ticks were disinfected on their surface with sequential washing in 10% sodium hypochlorite, tap water and distilled water. DNA was extracted with the QIAamp DNA Mini Kit (QIAGEN, Hilden, Germany) according to the manufacturer's instructions, including an overnight digestion in tissue lysis buffer and Proteinase K at 56 °C. An extraction control (tissue lysis buffer) was also processed in each set of tick samples to monitor cross-contamination.

PCR analyses (target genes, primers and cycling conditions) are summarized in Table 2. Frequently, the longer fragments of 18S and 28S rRNA genes were not successfully amplified, because of either negative results or aspecific PCR products. In such cases, amplification of a shorter part of the relevant gene was also attempted with different sets of primers (Table 2). The reaction mixture, in a volume of 25 µl, contained 1 U (0.2 µl) HotStarTaq Plus DNA polymerase, 2.5 µl 10 × CoralLoad Reaction buffer (including 15 mM MgCl_2_), 0.5 µl PCR nucleotide Mix (0.2 mM each), 0.5 µl (1 µM final concentration) of each primer, 15.8 µl ddH_2_O and 5 µl template DNA.

**Table 2:** Oligonucleotide sequences and cycle parameters of taxonomic PCRs used in this study

Target gene (approx. length)	Primers, probes (5'-3')	initial denaturation	cycle denaturation	cycle annealing	cycle extension	final extension	Cycle n =	References
cox1 (710 bp)	LCO1490 (GGT CAA CAA ATC ATA AAG ATA TTG G) HCO2198 (TAA ACT TCA GGG TGA CCA AAA AAT CA)	95 °C, 5 m	94 °C, 40 s	48 °C, 1 m	72 °C, 1 m	72 °C, 10 m	40	[24]
16S rRNA gene (460 bp)	16S + 1 (CTG CTC AAT GAT TTT TTA AAT TGC TGT GG) 16S-1 (CCG GTC TGA ACT CAG ATC AAG T)	95 °C, 5 m	94 °C, 40 s	51 °C, 1 m	72 °C, 1 m	72 °C, 10 m	40	[25]
18S rRNA gene (1300 bp)	NS1 (GTA GTC ATA TGC TTG TCT C) NS4a (GCC CTT CCG TCA ATT CCT TTA AG)	95 °C, 5 m	94 °C, 40 s	52 °C, 1 m	72 °C, 1 m	72 °C, 10 m	40	[17, 26]
18S rRNA gene (780 bp)	18S-F (CAT TAA ATC AGT TAT GGT TCC) 18S-R (CGC CGC AAT ACG AAT GC)	95 °C, 5 m	94 °C, 30 s	52 °C, 30 s 50 °C, 30 s 48 °C, 30 s 46 °C, 30 s	68 °C, 1 m	68 °C, 5 m	5 5 5 25	[27]
28S rRNA gene (700 bp)	28ScF (GTG GTA GCC AAA TGC CTC GTC ATC) 28SR (GAA TTC TGC TTC ACA ATG ATA GGA AGA GCC)	95 °C, 5 m	94 °C, 40s	58 °C, 1 m	72 °C, 1m	72 °C, 10 m	40	[28, 29]
28S rRNA gene (330 bp)	Tick-28S-C2-F (GCG GCG AGT AGG TCG GTA ACC) Tick-d9-D3-R (ACG TCA GAA TCG CTT CGG A)	95 °C, 5 m	95 °C, 30 s	60 °C, 30 s	72 °C, 1m	72 °C, 5 m	40	[30]

Reaction components: volume 25 µl, containing 1 U (0.2 µl) HotStarTaq Plus DNA polymerase, 2.5 µl 10 × CoralLoad reaction buffer (including 15 mM MgCl_2_), 0.5 µl PCR nucleotide Mix (0.2 mM each), 0.5 µl (1 µM final concentration) of each primer, 15.8 µl ddH_2_O and 5 µl template DNA

### Sequencing and phylogenetic analyses

In all PCRs, non-template reaction mixture served as negative control. Extraction controls and negative controls remained PCR negative in all tests. Purification and sequencing of the PCR products were done by Biomi Ltd. (Gödöllő, Hungary). Quality control and trimming of sequences were performed with the BioEdit program. Obtained sequences were compared to GenBank data by the nucleotide BLASTN program (https://blast.ncbi.nlm.nih.gov). New sequences were submitted to GenBank under the following accession numbers (cytochrome *c* oxidase subunit I [*cox*1] gene: OQ921940-OQ921984, 16S rRNA gene: OQ924680-OQ924707, 18S rRNA gene: OQ924736-OQ924748, 28S rRNA gene: OQ924930-OQ924948). Sequences from other studies (retrieved from GenBank) included in the phylogenetic analyses had nearly or exactly 100% coverage with sequences from this study. Therefore, although very few sequences of *Afrixodes* were previously deposited in GenBank, some of them had to be excluded because of their shortness (*Ixodes lemuris*: JX470176 [31]; *I. corwini*: AF113926 [32]). In addition, unpublished sequences of two *Ixodes* samples used in a previous study ([33]: *I. ricinus*, *I. trianguliceps*) were included to improve the phylogeny of *Ixodes* subgenera that are not present in South Africa. Sequence datasets were resampled 1000 times to generate bootstrap values. Phylogenetic analyses were conducted with the maximum likelihood method, Jukes-Cantor model (gamma distribution) with the MEGA version 7.0 software.

In addition, the sequences of both mitochondrial (*cox*1 and 16S rRNA) and nuclear (18S and 28S rRNA) genes (Additional file 1: Table S1) were aligned with the MAFFT algorithm [34] and then were concatenated in the Geneious Prime 2023.1.1 [35] software. The best fitting evolutionary model was selected by MEGA 11.0.10. A Bayesian consensus tree was created using the MrBayes [36, 37] in the Geneious Prime software. General time-reversible model was used to create the phylogenic tree with gamma distribution and invariant sites (GTR + G + I). The chain length was set to 10,000,000, sampling frequency to 5000 and burn-in length to 100,000. The gene partitions were treated as unlinked, and the random seed was set to 3504. The Bayesian tree was analyzed in the MEGA11 11.0.10 [38] software.

## Results

### Morphological identification and host associations of *Ixodes* species

**Fig. 1:**
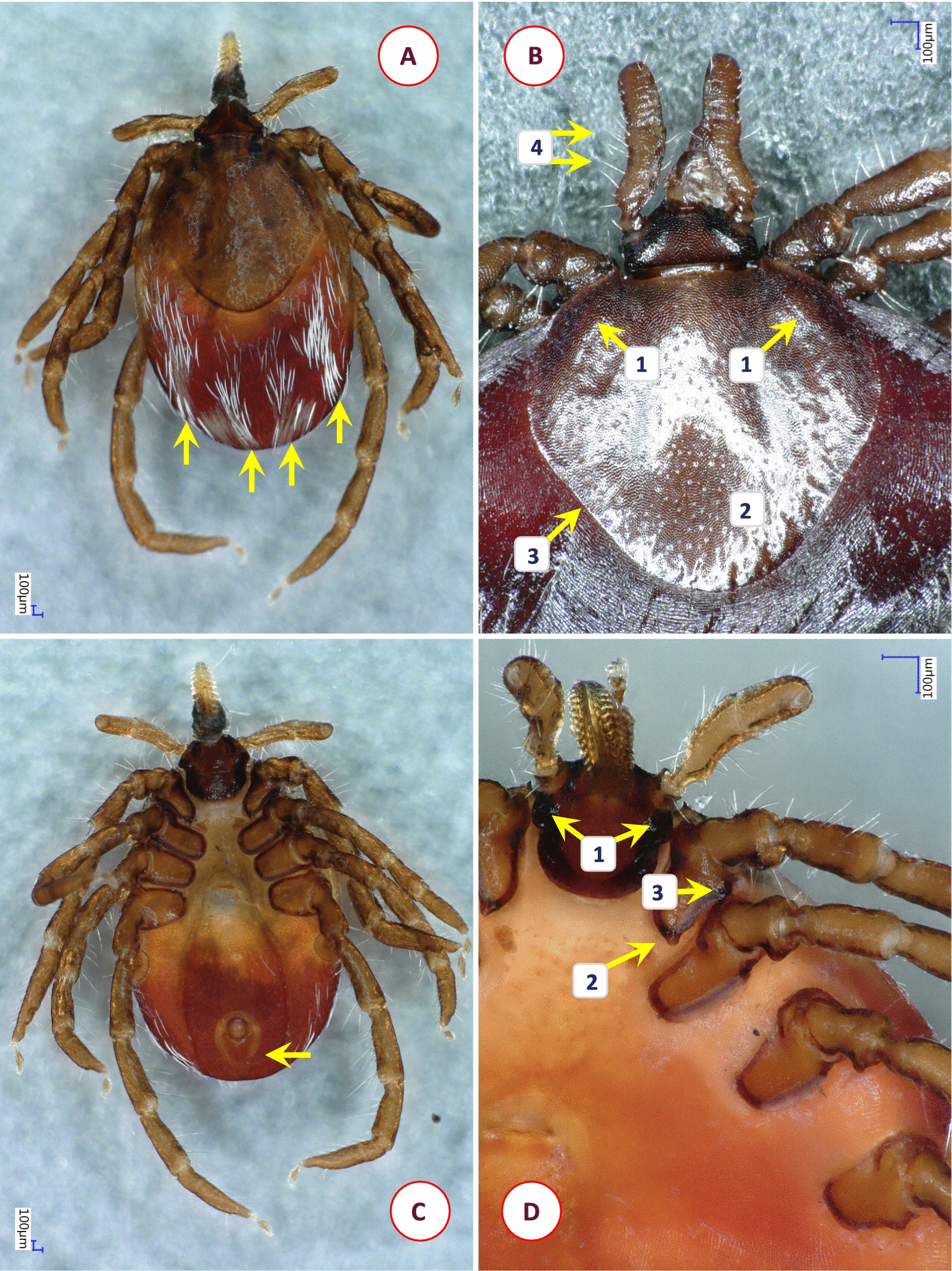
Key morphological characters of *Ixodes pilosus* group sp. I. female. **A** Dorsal view (arrows mark four stripes of bristles on the alloscutum). **B** Dorsal view of scutum (broader than long), basis capituli and palps (1—shorter lateral carinae; 2—scutum without hair, punctuation small-sized, dense; 3—posterolateral scutal margin slightly sinuous; 4—longest palpal hairs on segment II exceed the palpal diameter). **C** Ventral view (arrow marks anal groove which is short, converging). **D** Ventral view of basis and coxae (1—auriculae distinct, large, laterally rounded; 2—internal spur on coxae I short, distinct; 3—external spur on coxae I short, distinct)

**Fig. 2:**
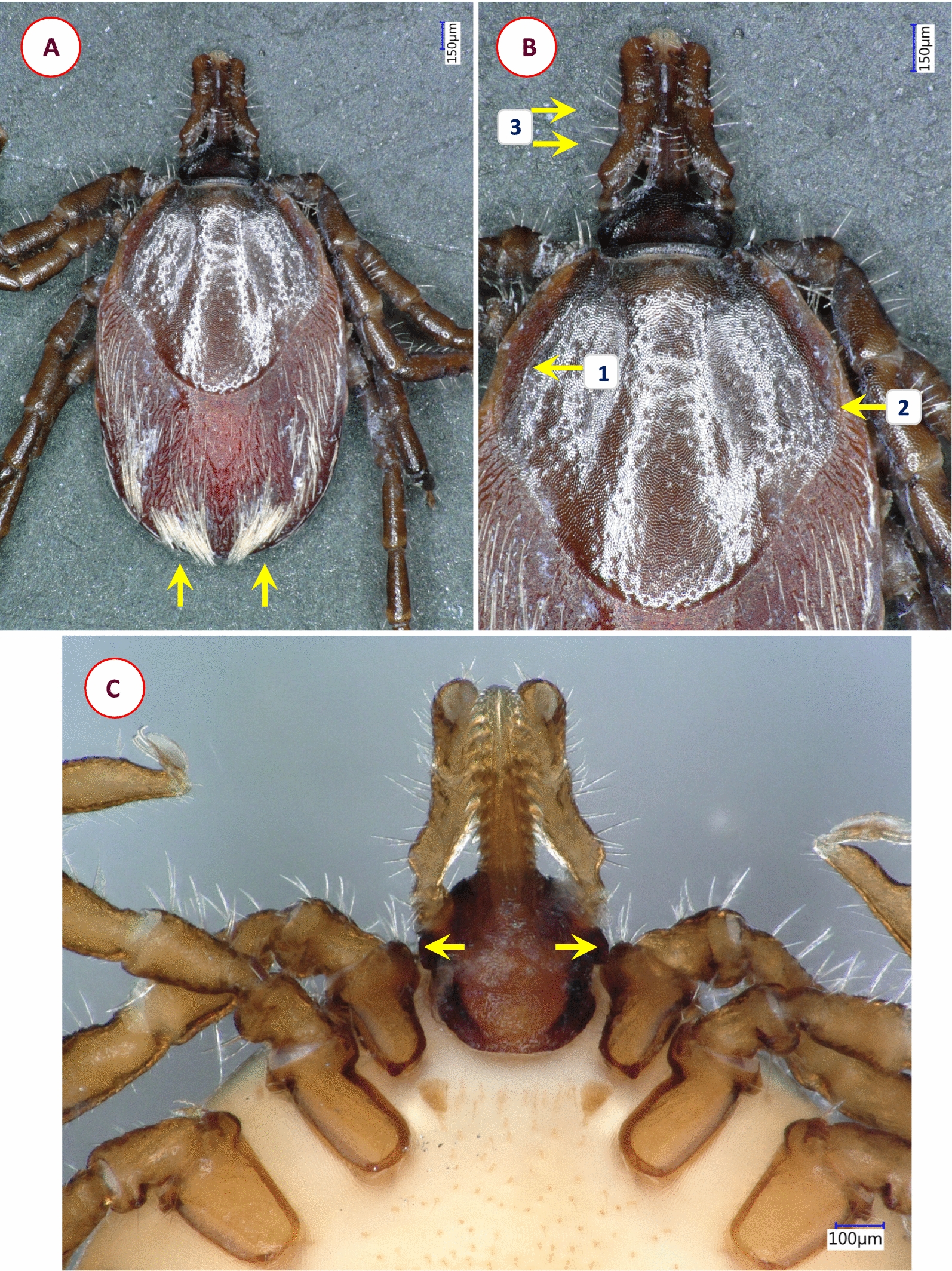
Key morphological characters of *Ixodes pilosus* group sp. II. female. **A** Dorsal view (arrows mark high density of alloscutal bristles posteriorly, forming tufts). **B** Dorsal view of scutum (longer than broad) and basis capituli (1—longer lateral carinae; 2—scutum, lateral margins parallel; 3—longest palpal hairs on segment II exceed the palpal diameter). **C** Ventral view (arrows mark auriculae as distinct, large, laterally flattened)

**Fig. 3:**
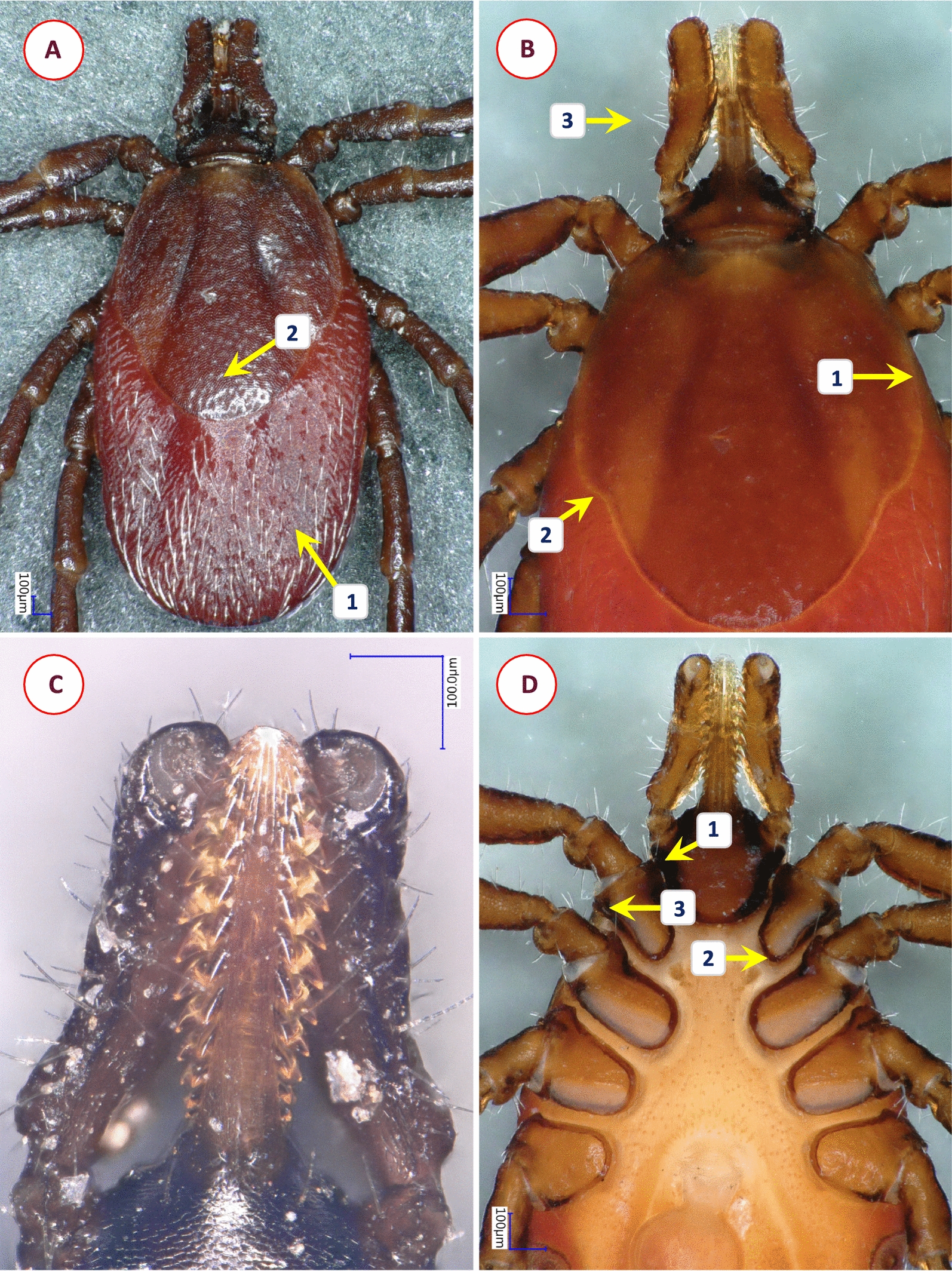
Key morphological characters of *Ixodes* cf. *rubicundus* female. **A** Dorsal view (1—evenly distributed bristles on alloscutum; 2—scutum without hair, punctuation large-sized, dense). **B** Dorsal view of scutum and basis capituli (1—scutum elongated with parallel sides, length to width ratio 1.2; 2—posterolateral scutal margin distinctly sinuous; 3—palpal hairs shorter than palpal diameter). **C** Hypostome (dental formula: at the tip 4/4, two or three rows of 3/3 and seven rows of 2/2 teeth). **D** Ventral view of basis and coxae (1—auriculae indistinct; 2—internal spur on coxae I absent; 3—external spur on coxae I small)

**Fig. 4:**
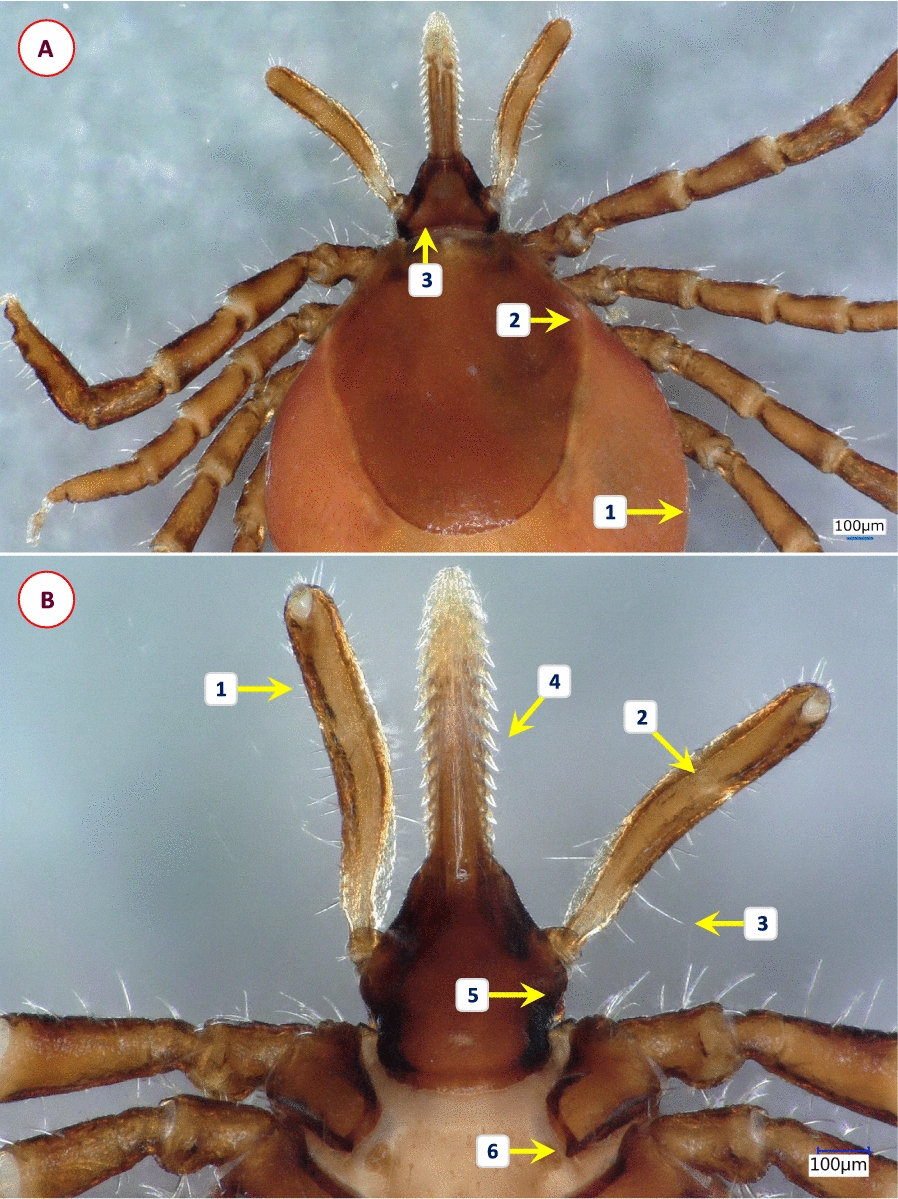
Key morphological characters of *Ixodes ugandanus* female. **A** Dorsal view of scutum and basis capituli (1—short, rounded body; 2—scutum posteriorly broad but widest well in front of midlength; 3—areae porosae subtriangular, distance between them less than their width). **B** Ventral view of basis and first coxae (1—palps long, their sides parallel along anterior straight part, lateral margins concave; 2—separation of segments II-III indistinct; 3—palpal hairs longest close to base of segment II; 4—lateral teeth on hypostome long and strong; 5—auriculae indistinct; 6—internal spur on coxae I pointed, medially directed)

**Fig. 5:**
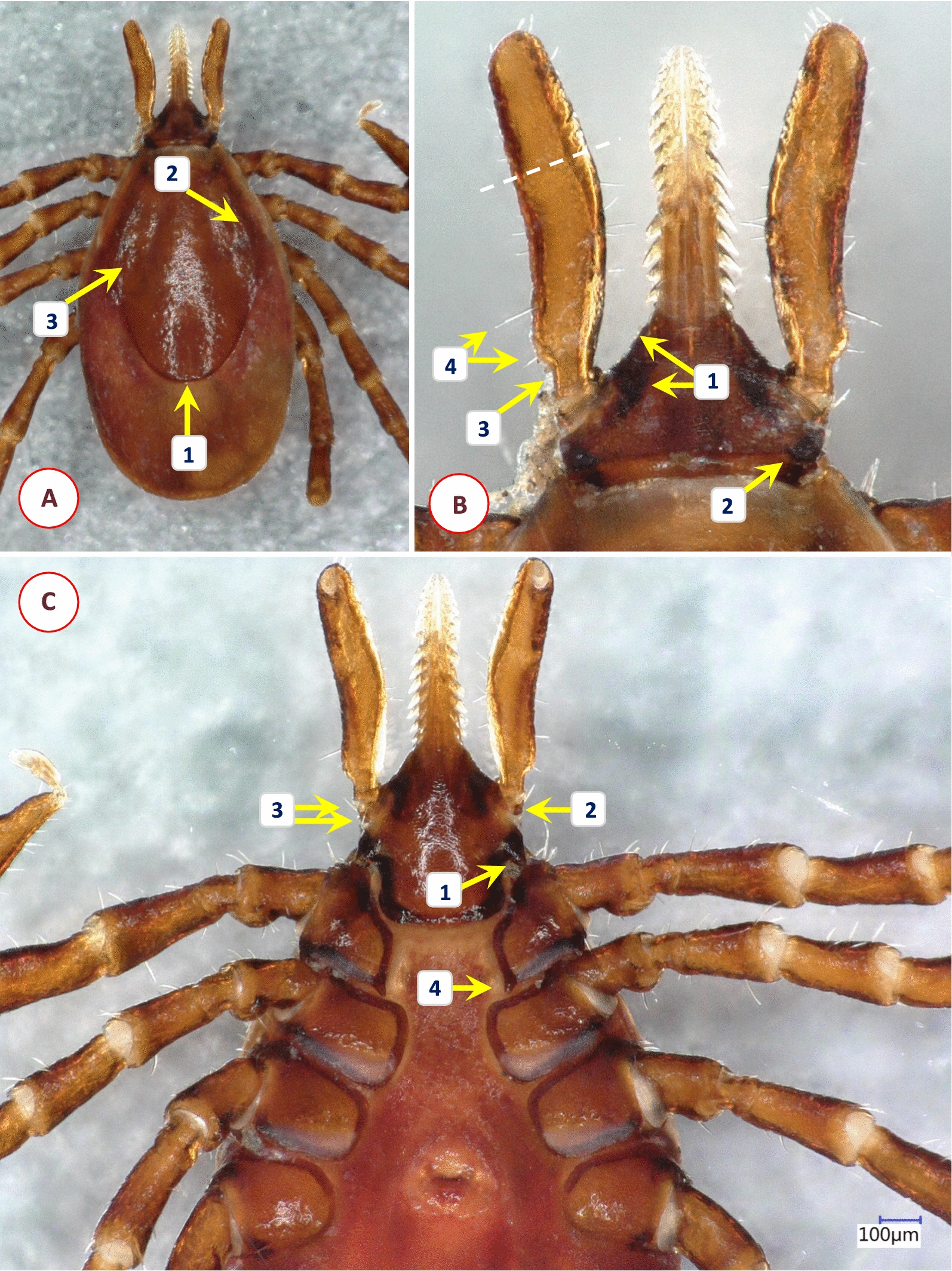
Key morphological characters of *Ixodes nairobiensis* female. **A** Dorsal view (1—scutum rhomboidal, tapering to rounded posterior end, length to width ratio approx. 1.5; 2—lateral carinae straight; 3—cervical grooves shallow but broad). **B** Dorsal view of basis capituli (1—basis capituli triangular, its anteriolateral edge straight, supported from behind by a caudally tapering dark area of sclerotization; 2—small, rounded, caudally directed cornuae; 3—palps "stalked", approximately four times as long as broad at their maximum width, ratio of length of palpal segment II to III approximately 1.7; white dashed line indicates indistinct separation of segments II-III; 4—hair longest posteriorly on palpal segment II, one short hair on its caudolateral protuberance). **C** Ventral view of basis and coxae (1—well-developed, tapering and pointed auriculae, directed posterolaterally; 2—palpal segment I with flange-like extension and (3) two hairs; 4—coxa I with caudomedially directed spur, not in line with medial edge of coxa I)

**Fig. 6:**
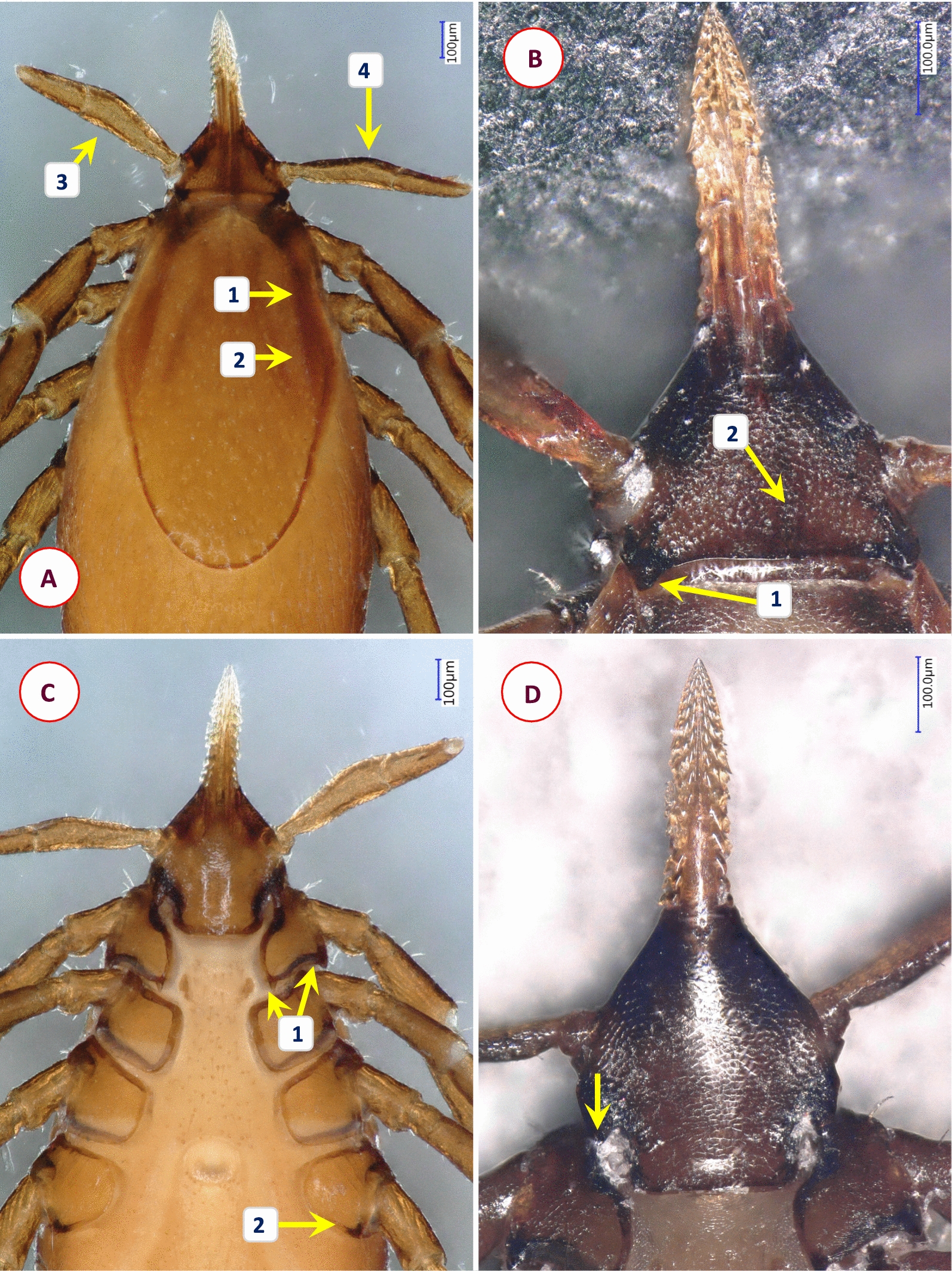
Key morphological characters of *Ixodes rhabdomysae* female. **A** Dorsal view (1—scutum rhomboid, with lateral carinae as ridges; 2—cervical grooves broad, shallow, lightly colored; 3—lateral margin of palps straight; 4—medial edge of palpal segment II with obtuse-angled convexity). **B** Dorsal view of basis capituli (1—cornuae sharp, caudally directed; 2—areae porosae only slightly depressed, delimited medially by a dark ridge). **C** Ventral view of basis and coxae (1—coxae I with small internal and prominent external spurs; 2—coxae IV with distinct external spur). **D** Ventral view of basis (arrow marks sharp, caudally directed auricula)

**Fig. 7:**
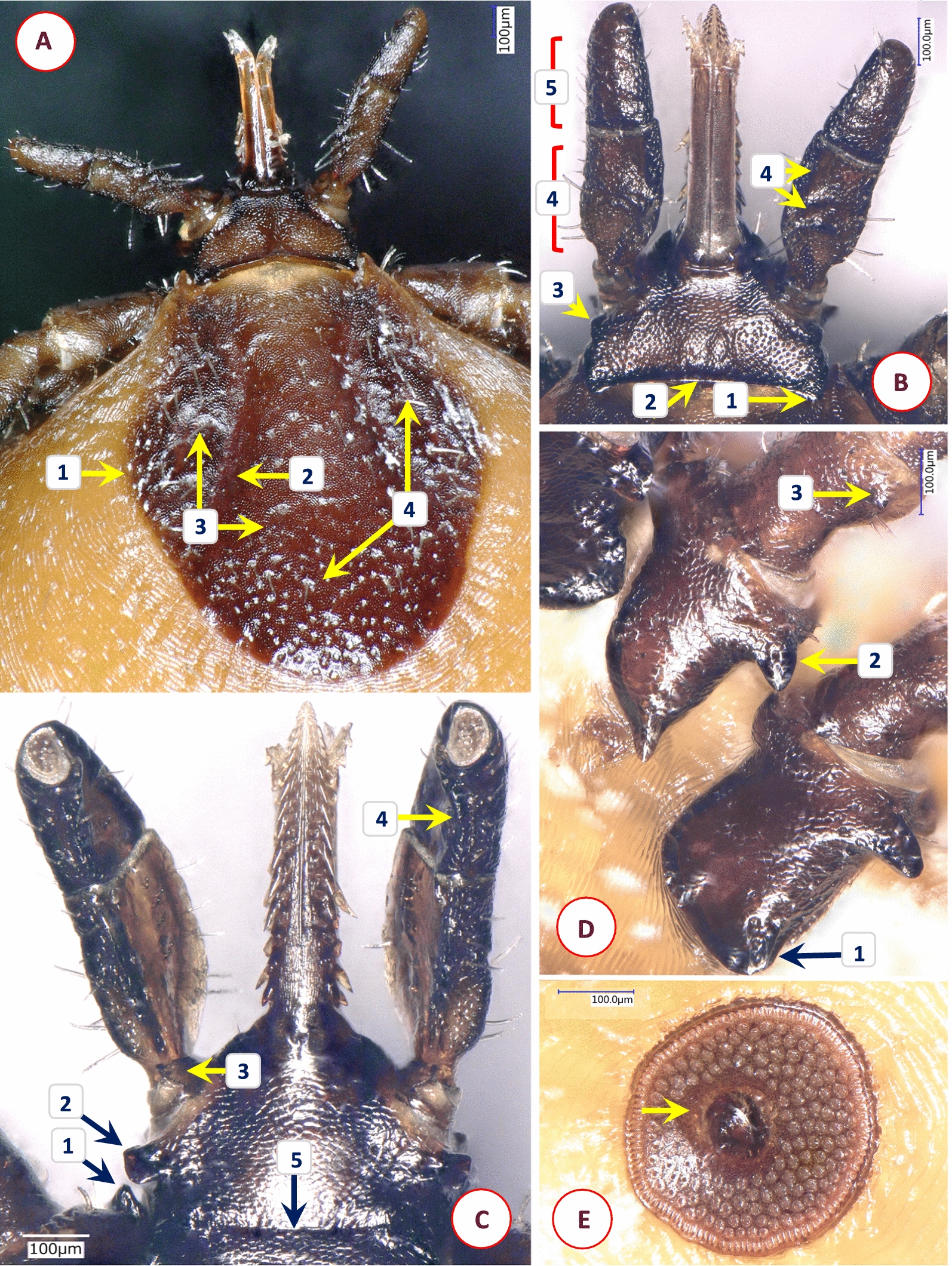
Key morphological characters of *Ixodes theilerae* female. **A** Morphology of scutum (1—scutum widest slightly anterior to midlength; 2—cervical grooves broad, shallow; 3—surface finely punctuate, with lateral rugosities; 4—bristles (hair covering) in lateral fields and mid-region of scutum). **B** Dorsal view of basis capituli and palps (1—cornuae short posterolateral projections; 2—posterior margin slightly concave; 3—basis laterally undulate, with anteriolateral protuberance (beneath which the auriculae are visible); 4—palpal segment II with longitudinal and transverse groove, outer profile irregular; 5—palpal segment III outer profile straight). **C** Ventral view of basis capituli and palps (1—scapulae sharp, pointed; 2—auriculae terminate in sharp, straight edge; 3—palpal segment I with mesoventral, plate-like projection; 4—ventral edge of palpal segment III sinusoid; 5—transverse groove behind auriculae, at the level of "waist"). **D** Coxae I-II and trochanter I (1—coxa II with thickened posterointernal margin; 2—large external spur on coxae I-IV; 3—trochanteral spurs (I-III) short). **E** Spiracle opening (subcircular, macula excentric, surrounded by a “C” chape area void of aeropyles, the latter in 2–5 rows)

**Fig. 8:**
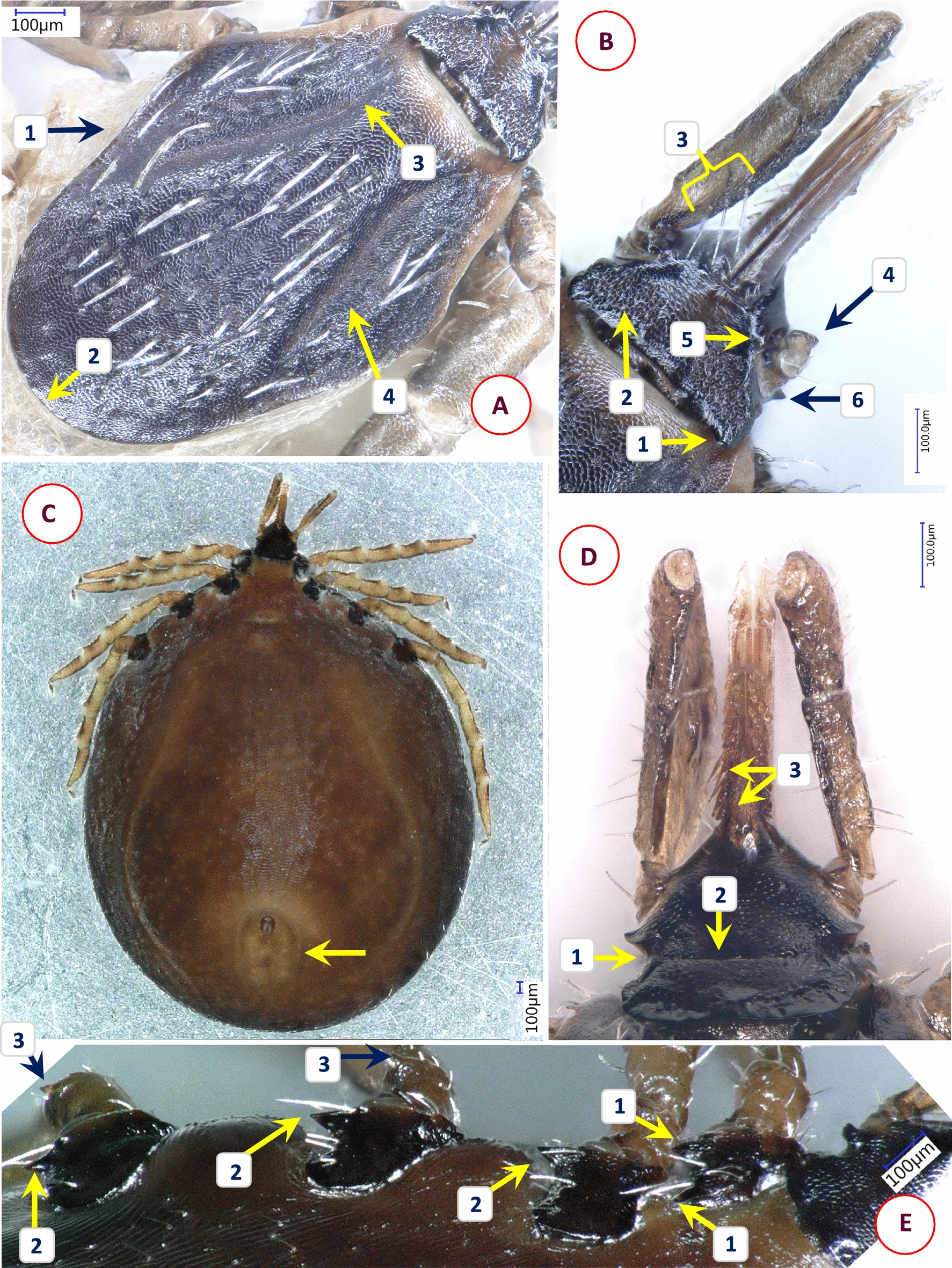
Key morphological characters of *Ixodes* cf. *daveyi* female. **A** Morphology of scutum (1—posterolateral edge of scutum with concavity behind maximum breadth; 2—posterior scutal margin rounded; 3—cervical grooves deep, convergent in front, divergent posteriorly; 4—surface of scutum finely punctate, with rugose lateral fields). **B** Dorsal view of basis capituli and palp (1—cornuae rounded, backwardly projecting protuberances; 2—porose areas very large, subtriangular; 3—basal medial edge of palpal segment II with three long bristles; 4—palpal segment I with ventral protrusion; 5—anteriolateral margin of basis capituli forms a ridge behind palpal basis; 6—auriculae angular, trenchant). **C** Ventral view (arrow marks horseshoe-shaped anal groove). **D** Ventral view of basis (1—basis strongly constricted behind auriculae, posterior width similar to that of auriculae; 2—transverse groove behind auriculae, at the level of "waist"; 3—hypostome with fine denticles and broad median unarmed surface). Note that the palp on the right is broken at the base of segment II. **E** Lateral view of coxae, trochanters (1—coxa I with sharply pointed internal and broad, tapered external spur; 2—coxae II-IV with external spurs, sharp when viewed laterally (decreasing in this order); 3—trochanters with distal spurs as sharp protrusions)

**Fig. 9:**
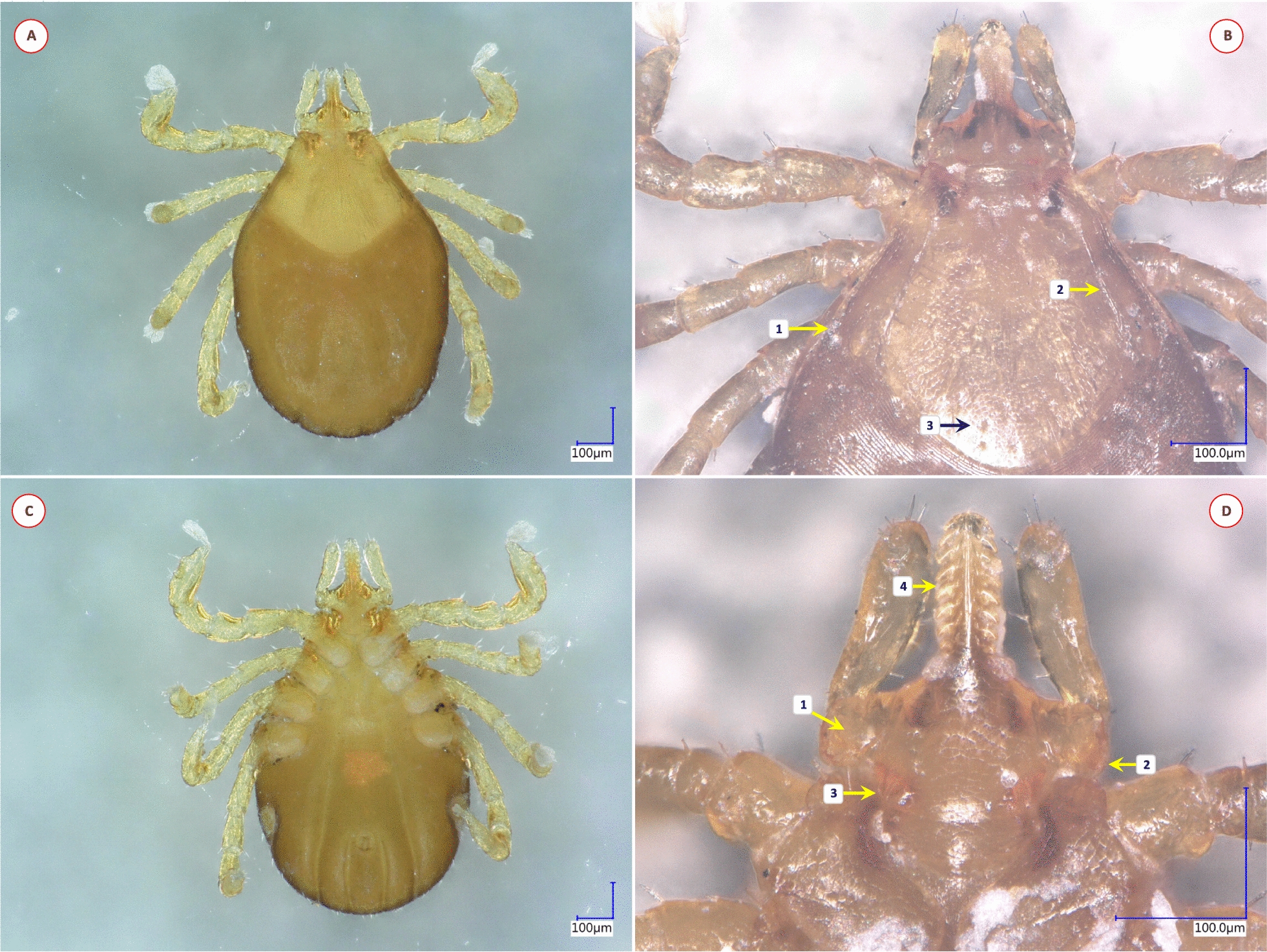
Key morphological characters of *Ixodes alluaudi* nymph. **A** Dorsal view. **B** Dorsal view of scutum and basis capituli (1—scutum broadest at mid-length; 2—cervical grooves lacking, lateral carinae straight; 3—surface finely punctate). **C** Ventral view. **D** Ventral view of basis capituli and hypostome (1—palpal segment I and the base of segment II overlapped by chitinous extension; 2—posterolateral angle of basis rounded; 3—auriculae pointed; 4—hypostome with 7 rows of 2/2 denticles)

**Fig. 10:**
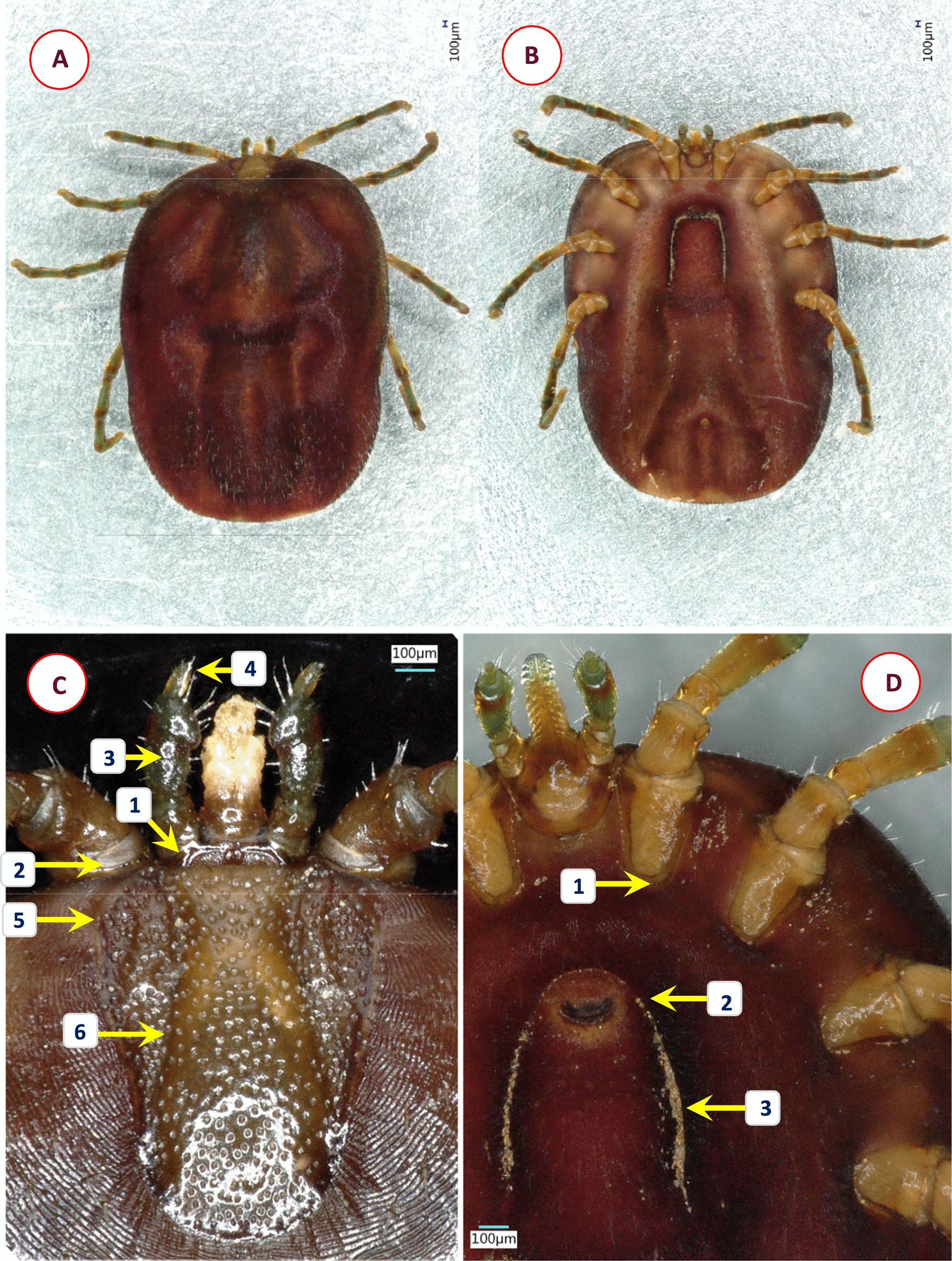
Key morphological characters of *Ixodes uriae* female. **A** Dorsal view. **B** Ventral view. **C** Scutum, basis capituli and palps (1—basis small and short, with large areae porosae; 2—the rim of coxa I joining trochanter I visible dorsally as a broad ring; 3—palpal surface smooth and shining; 4—palpal segment IV prominent from above; 5—scutum long, widest at its front; 6—cervical grooves well-defined, surface of scutum with numerous punctuations). **D** Ventral view of genital pore and coxae (1—no internal [and external] spur on coxae; 2—genital aperture between 2nd intercoxal space; 3—genital grooves anteriorly subparallel)

Based on morphological characters, 11 different species were identified among the 58 *Ixodes* specimens analyzed in this study. Their most important diagnostic characters are shown in Figs. 1, 2, 3, 4, 5, 6, 7, 8, 9 and 10. The majority of adult ticks belonged to the *I. pilosus* group (*n* = 20) with two species (Table 3) and a species (*Ixodes* cf. *rubicundus*) most similar to *I. rubicundus* but having a small external spur on coxa I (*n* = 18) (Fig. 3). In addition, single specimens of *Ixodes rhabdomysae*, *I. ugandanus* and *I. nairobiensis* were also found. On the other hand, only subadults (mostly nymphs) infested other mammalian hosts, as exemplified by *Ixodes simplex* (*n* = 1) and *I. alluaudi* (*n* = 3) (Table 1). Considering bird-infesting ticks, in decreasing order of the number of their samples, these belonged to *Ixodes theilerae* (*n* = 7), *I. uriae* (*n* = 4) and a species most similar to *I. daveyi*, provisionally named *I.* cf. *daveyi* (*n* = 2).

**Table 3:** Comparative morphology of *Ixodes pilosus* group sp. I and sp. II. Asterisk (*) marks those characters based on which they are different

Character	*Ixodes pilosus* group sp. I	*Ixodes pilosus* group sp. II
Alloscutal setae (*)	In four stripes	High density posteriorly, forming tufts
Setae (*)	Thick	Thin
Scutal shape (*)	Broader than long	Longer than broad
Scutal length/width ratio (*)	0.93	1.05
Lateral carinae (*)	Short	Long
Scutal surface	Without hair, punctuation small-sized, dense	Without hair, punctuation small-sized, dense
Lateral scutal margin (*)	Curved	Relatively straight, parallel
Posterolateral scutal margin (*)	Slightly sinuous	Moderately sinuous
Maximum palpal hair length on segment II	Exceeding palpal diameter	Exceeding palpal diameter
Auriculae (*)	Large, laterally rounded	Large, laterally flattened
Internal spur on coxae I	Small, distinct	Small, distinct
External spur on coxae I	Small, distinct	Small, distinct

Regarding domestic dogs and wild living carnivores, two species of the *I. pilosus* group, as well as *I.* cf. *rubicundus*, predominated on these hosts, but two additional species, *I. ugandanus* and *I. nairobiensis*, were also identified (Table 1). *Ixodes* cf. *rubicundus* was also collected from Southern African hedgehogs (*Atelerix frontalis*). *Ixodes alluaudi* was only removed from lesser gray-brown musk shrews (*Crocidura silacea*) and single specimens of *I. simplex* and *I. rhabdomysae* from Natal long-fingered bat (*Miniopterus natalensis*) and brown greater galago (*Otolemur crassicaudatus*), respectively. Three further *Ixodes* species were exclusively associated with avian hosts; in particular, *I. uriae* was collected from southern rockhopper penguins (*Eudyptes chrysocome*) and two species from passeriform birds: *I. theilerae* from five weaver species (family Ploceidae) and *I.* cf. *daveyi* from two species of robin-chats (family Muscicapidae).

### Molecular analyses of *Ixodes* species

Considering *cox*1 sequences, specimens collected in this study and named *I. pilosus* group sp. I. had low genetic diversity, with 99.5–100% (632–635/635 bp) sequence identity to each other. The *cox*1 sequence of the species named *I. pilosus* group sp. II (OQ921974) had only 86.8% (551/635 bp) sequence identity compared with *I. pilosus* group sp. I. (OQ921940). The only *I. pilosus cox*1 sequence available in GenBank (GU437874) also had low, i.e. 90.1% (530/588 bp) and 85.4% (502/588 bp), sequence identity with these species of the *I. pilosus* group from this study, respectively. *Ixodes* cf. *rubicundus* had a higher degree of *cox*1 sequence identity in this study than *I. pilosus*, namely 99.8–100% (635–636/636 bp). Compared with GenBank data, this species (corresponding to sequence OQ921949) had only 88.4% (563/637 bp) sequence identity with a sequence available in GenBank (KY457530) and assigned to *I. rubicundus* from South Africa (Fauresmith). Based on GenBank data, the *cox*1 sequence of *I. rhabdomysae* (OQ921961) showed the highest [92.4% (535/579 bp)] sequence identity to the corresponding sequence of *Ixodes bakeri* (GU437873). *Ixodes ugandanus* collected in this study had the highest [87.9% (507/577 bp)] sequence identity with *I. rasus* reported from Cameroon (OP718638).

Among bird-associated tick species, the two *cox*1 sequences of *I. theilerae* had 1 bp difference from each other. According to *cox*1 sequences from GenBank, one of them (OQ921964) was most similar (92%, 585/636 bp) to the “B” mitochondrial lineage of *Ixodes frontalis* reported from Hungary (KU170508). Similarly, the *cox*1 sequence of *I.* cf. *daveyi* (OQ921973) had the highest level (91%, 566/622 bp) of identity to the same sequence of *I. frontalis*. Finally, based on the *cox*1 gene, *I. uriae* showed 99.5–99.8% (636/639–639/640 bp) intraspecific genetic identity within Marion Island but had only 96.4% (614/637 bp) sequence identity with *I. uriae* reported from Canada (KX360345).

The 16S rRNA sequences of *I. pilosus* group sp. I. were 99.7–100% (380–381/381 bp) identical to each other and 98.7% (377/382 bp) identical to the only sequence available in GenBank for this species (AF113927). At the same time, the 16S rRNA sequences of *I. pilosus* group sp. I. were only 89% (339/381 to 333/374 bp) identical to those of *I. pilosus* group sp. II analyzed here from South Africa. Based on 16S rRNA gene, the heterogeneity within *I.* cf. *rubicundus* was higher than in the case of *I. pilosus*: the sequences were 99.2–100% (372–375/375 bp) identical to each other. Moreover, similarly to the *cox*1 gene, ticks identified morphologically as *I.* cf. *rubicundus* in this study showed only 87.4% (334/382 bp) sequence identity compared with the sequence deposited in GenBank under the name *I. rubicundus* (KY457530, from South Africa, Fauresmith). The 16S rRNA gene sequences of *I. rhabdomysae* (OQ942703), *I. nairobiensis* (OQ942705) and *I. ugandanus* (OQ942730) showed the highest [90.6% (345/381 bp), 92.3% (374/405) and 93.1% (353/379 bp)] sequence identity to the corresponding sequence of *I. rasus* reported from Cameroon (OP698035). The 16S rRNA sequence of *I. simplex* in this study (OQ942713) had 99.5% (384/386 bp) identity to both corresponding sequences of the same species also reported from South Africa (KY457531, KY457532) and lower [98.4% (380/386 bp)] similarity to *I. simplex* from Europe (Hungary: KM455970).

Among bird-associated tick species, the three *I. theilerae* sequences were nearly (99.7–100%: 378–379/379 bp) identical to each other but differed in 4% (15/375 bp) from *I.* cf. *daveyi* (OQ942719 vs OQ942720) in their 16S rRNA gene. The four sequences of *I. uriae* differed only in 1 bp (99.7–100% identity) among samples collected in this study on Marion Island, and also when compared in a small geographical scale with a sample from the Antarctica (D88304), but only 96.9% (369/381 bp) identical to a conspecific sequence reported from Magdalena Island, Chile (MK570083).

### Phylogenetic analyses of *Ixodes* species

**Fig. 11:**
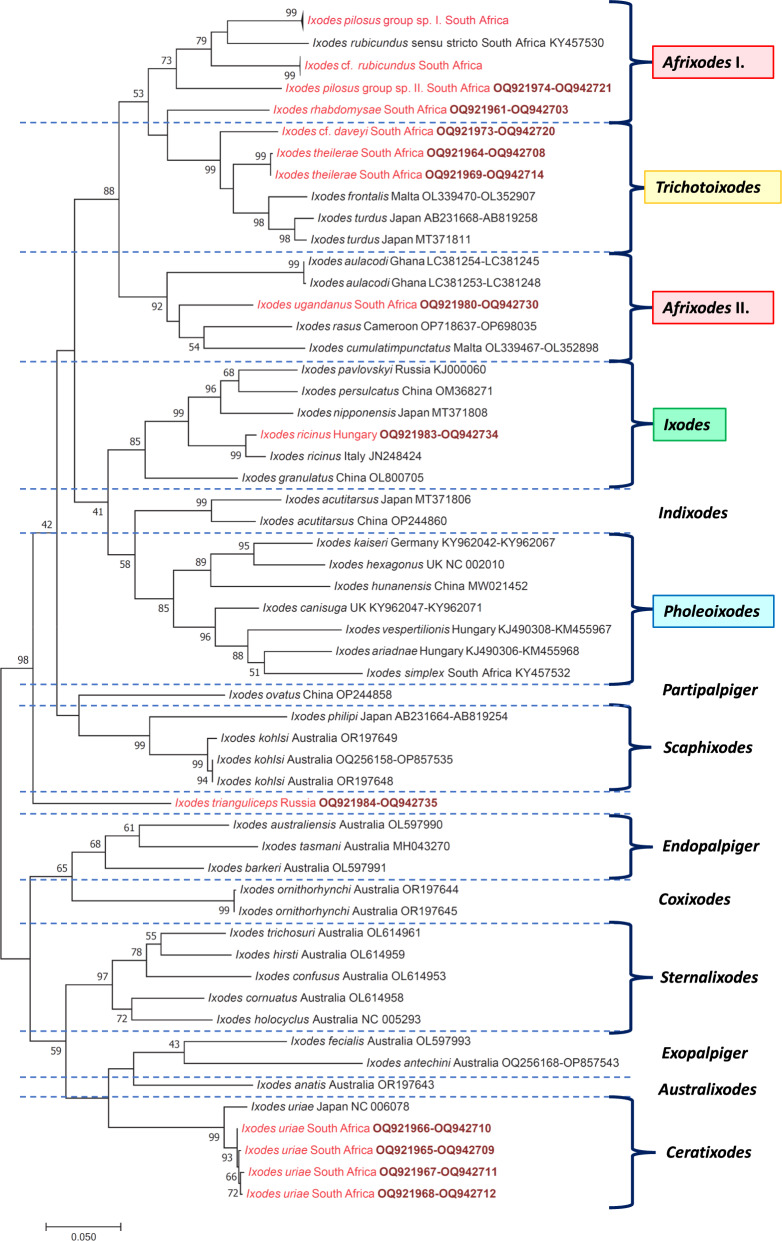
Phylogenetic tree based on concatenated sequences of the *cox*1 and 16S rRNA genes, focusing on Old World *Ixodes* species. In each row of individual sequences, the country of origin and the GenBank accession number are shown after the species name. Sequences from this study are indicated with red fonts and bold, maroon accession numbers. *Ixodes pilosus* group sp. I. and *I.* cf. *rubicundus* are represented by multiple sequences (Table 1), and their branches are shown collapsed. The evolutionary history was inferred by using the maximum likelihood method based on the general time-reversible model. The tree is drawn to scale, with branch lengths measured in the number of substitutions per site. The analysis involved 74 nucleotide sequences. All positions containing gaps and missing data were eliminated. There were a total of 892 positions in the final dataset. Evolutionary analyses were conducted in MEGA7

Based on concatenated *cox*1 and 16S rRNA gene sequences, *I.* cf. *daveyi* and *I. theileriae* clustered together with Palearctic members of the subgenus *Trichotoixodes* (i.e. *I. frontalis* and *I. turdus*) (Fig. 11). All four *Trichotoixodes* belonged to a monophyletic group with high (99%) support (Fig. 11). Phylogenetic analysis of 16S rRNA gene sequences showed that *I. simplex* belonged to the same clade with its representative from Europe (Additional file 2: Fig. S1). Based on its 16S rRNA gene, *I. alluaudi* was phylogenetically most closely related to *I. antechini* (subgenus *Exopalpiger*) (Additional file 2: Fig. S1). *Ixodes uriae* from Southern Africa, Marion Island, clustered with conspecific sequences with different, large-scale geographical origin (Additional file 2: Fig. S1).

**Fig. 12:**
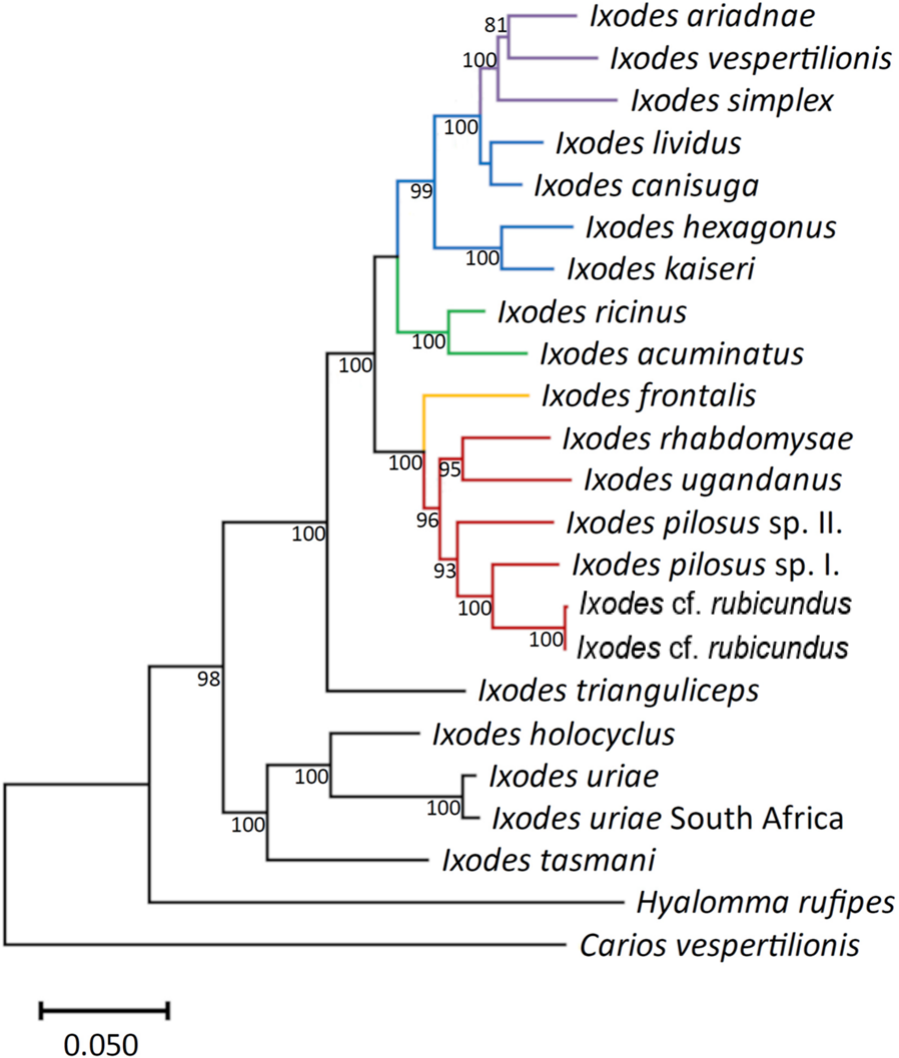
Concatenated phyltree based on two mitochondrial (*cox*1 and 16S rRNA) and two nuclear (18S and 28S rRNA) genetic markers. The sequences of four genes were aligned with the MAFFT algorithm and then were concatenated (in the above order) in the Geneious Prime 2023.1.1 software. A Bayesian consensus tree was created using the MrBayes in the Geneious Prime software. General time-reversible model was used to create the phylogenic tree with gamma distribution and invariant sites (GTR + G + I). The chain length was set to 10,000,000, sampling frequency to 5000 and burn-in length to 100,000. The gene partitions were treated as unlinked, and the random seed was set to 3504. The Bayesian tree was analyzed in the MEGA11 11.0.10 software

Regarding the relationships among *Ixodes* subgenera, in both the concatenated *cox*1 and 16S rRNA gene tree (Fig. 11) and the concatenated mitochondrial-nuclear marker tree (Fig. 12), the subgenus *Afrixodes* was most closely related to subgenus *Trichotoixodes*, the latter containing exophilic bird-associated tick species. Based on the former, *Afrixodes* was a monophyletic clade only if including *Trichotoixodes* (Fig. 11). The monophyletic group gathering *Afrixodes* and *Trichotoixodes* was also confirmed with high bootstrap value when using both mitochondrial and nuclear genetic markers in a concatenated tree (Fig. 12). Interestingly, subgenera *Ixodes* and *Pholeoixodes* belonged to a sister group to *Afrixodes* and *Trichotoixodes* in the concatenated mitochondrial as well as in the mitochondrial-nuclear gene-based phylogenetic trees (Figs. 11 and 12). In both of these analyses, *I. trianguliceps* was the sister clade to the group including *Pholeoixodes*, *Ixodes*, *Afrixodes* and *Trichotoixodes*, whereas Australasian *Ixodes* species formed a sister group to all other *Ixodes* species (based on mitochondrial-nuclear sequences receiving high, 98% support) (Figs. 11 and 12).

## Discussion

This is the first phylogenetic study to our knowledge that involves subgenera *Afrixodes* and *Trichotoixodes* with multiple species and both mitochondrial and nuclear genetic markers. In previous large-scale analyses, either the subgenus *Trichotoixodes* was omitted [19, 32] or *Afrixodes* was represented by a single species [39] or only a single mitochondrial marker was targeted for a low number of species [40]. In other words, only a few African *Ixodes* species had available sequences in GenBank, especially from the subgenus *Afrixodes* (i.e. *Ixodes pilosus*, *I. bakeri*, *I. fynbosensis*: [7]; *I. aulacodi*: [41], *I. lemuris*: [31]). The complete mitogenome is also available for a few species (e.g. *I. rubicundus*: [19]). Optimally either the latter or multiple (simultaneously amplified two mitochondrial or both mitochondrial and nuclear) genetic markers were lacking in databases. This is to some extent compensated by the present study, in which up to four genetic markers are provided for 11 South African *Ixodes* species.

All ticks identified to the species level in this study were already known to occur in South Africa [8, 11, 12]. Considering the so-called *I. pilosus* group within the subgenus *Afrixodes*, it was proposed earlier on a morphological basis that it includes at least three different species [10, 11]. This was confirmed here with molecular-phylogenetic methods, i.e. *I. pilosus* group species I and II, which are illustrated and barcoded here, had *cox*1 sequence difference > 10% compared with each other and a third member of this group already available in GenBank, thus exceeding the level of average interspecific sequence divergence (6.1%) reported for this genetic marker in case of ticks [42]. Based on its morphological description, *I. pilosus* group sp. II corresponds to the type described by Arthur [8], and sp. I may represent another species known but not yet named [11]. Interestingly, sequences from the specimens identified morphologically in this study as *I.* cf. *rubicundus* were also significantly different from the GenBank entry under this name, probably representing a new species.

Ticks infesting passeriform (song) birds in this study were morphologically identified as *I. theilerae* and *I.* cf. *daveyi*. As already reported [8], *I. daveyi* might also occur in southern Africa. *Ixodes daveyi* was regarded as a member of subgenus *Trichotoixodes* already by Reznik [43]; therefore, the presence of syncoxae (I-III) as mentioned by Arthur [8], but not originally by Nuttall [20], is probably a mistaken attribute. Arthur also mentions the hairless surface of scutum of *I. daveyi* with a question mark, but since other species of *Trichotoixodes* have (frequently prominent) hair covering on the scutum, as exemplified by *I. frontalis* (syn. *I. pari*) and *I. theilerae* in Africa [8]. This is likely a character of *I. daveyi* as well, as shown here for *I.* cf. *daveyi* (Fig. 8). Since one of the bird species from which *I.* cf. *daveyi* was removed, the Cape robin-chat (*Cossypha caffra*), occurs up to the northern latitude of Sudan and Uganda and in east Africa [44], it is likely that *I.* cf. *daveyi* might infest this bird species throughout its geographical range. Thus, although *I. daveyi *sensu stricto typically occurs north of South Africa, its transportation by birds into the latter region seems to be possible, and it may rarely infest hosts other than birds as already reported [8]. *Ixodes spinae* was also reported in South Africa from Southern red bishop (*Euplectes orix*) [45] on which *I.* cf. *daveyi* was found in this study, and similarly to *I. daveyi* this tick species was also reported from hyraxes (*Procavia capensis*) [8], raising the possibility that these two species might be confused. However, the presence of *I. spinae* in the study material was excluded based on the shape of its scutum, cervical grooves and palps (as shown in [8]). Similarly, *Ixodes domerguei* was excluded because of the presence of trochanteral spurs on ticks collected from birds in the present study [21, 22].

All tick species collected in this study from domestic or wild carnivores had previously reported host associations [11, 46], except for the African civet (*Civettictis civetta*) and the marsh mongoose (*Atilax paludinosus*), which appear to be new host records for *I. ugandanus* and *I. nairobiensis*, respectively. Considering insectivores, *I. rubicundus*-like ticks are newly reported from the southern African hedgehog (*Atelerix frontalis*) as it was not even mentioned among the hosts of *I. rubicundus *sensu stricto [11]. *Ixodes alluaudi* was already reported from the greater red musk shrew (*Crocidura flavescens*) [8], but not the lesser gray-brown musk shrew (*C. silacea*), as in this study. Regarding bats and primates, Natal long-fingered bat (*M. natalensis*) appears to be a new host record for *I. simplex* as well as the brown greater galago (*Otolemur crassicaudatus*) for *I. rhabdomysae* [11].

According to the present results, *I. theilerae* is frequently associated with weavers (Ploceidae), confirming previous data [11]. Nevertheless, this tick species was newly collected from yellow-crowned bishop (*Euplectes afer*). This study also provides a new host record of bird ticks, i.e. robin-chats (*Cossypha* spp.), for *I.* cf. *daveyi*. Although the occurrence of *I. daveyi* in South Africa was believed to be uncertain [12], one of the avian hosts from which the similar *I.* cf. *daveyi* was collected in this study, the Cape robin-chat (*Cossypha caffra*), was formerly reported to carry an *Ixodes* specimen not identified to the species level [45]. In the present study, *I. uriae* was collected from Southern rockhopper penguin (*E. chrysocome*), a long-recognized host of this tick species [8, 47].

Considering the overall phylogenetic relationships of *Ixodes* subgenera, the topology of both concatenated phylogenetic trees (based on mitochondrial or on both mitochondrial and nuclear markers) confirmed that there are two distinct phylogenetic clades (lineages) of prostriate ticks, i.e. the Australasian and all other *Ixodes* species [6]. Subgenera *Ixodes* and *Pholeoixodes* clustered in the sister group to the clade of *Afrixodes*-*Trichotoixodes* based on both mitochondrial and mitochondrial-nuclear markers, in part similar to previous results [48].

Importantly, based on the nuclear 18S rRNA gene, it was reported that the Palearctic *I. trianguliceps* belongs to a sister group of the clade of subgenera *Ixodes* and *Pholeoixodes* [49]. However, when including *Afrixodes* and *Trichotoixodes*, based on mitochondrial markers, it occupied a basal position to all these subgenera, well separated from subgenus *Exopalpiger* where it was formerly thought to belong [3]. Thus, the taxonomic position of *I. trianguliceps* should be revised at the subgenus level.

Concerning African species of this study, the results of molecular-phylogenetic analyses reflected well the taxonomic positions assigned historically on morphological bases: *I. rhabdomysae*, *I. ugandanus* and *I. nairobiensis* belonged to the cluster of subgenus *Afrixodes* and *I. theilerae*, *I.* cf. *daveyi* to the subgenus *Trichotoixodes*. The subgenus *Trichotoixodes* Reznik, 1961, was created to comprise *Ixodes* species typically associated with avian hosts in all developmental stages, sharing common morphological characters such as the presence of truncated auriculae in the female and external spur on all coxae [43]. The nine species currently allocated into this subgenus have somewhat allopatric global distribution, i.e. *Ixodes brunneus* being Nearctic, *I. silvanus* and *I. copei* Neotropical, *I. frontalis* and *I. turdus* Palearctic. Within the Afrotropical region, *Ixodes daveyi* and *I. euplecti* occur predominantly in central-northern Africa, whereas *I. theilerae* in southern Africa and *I. domerguei* in Madagascar [2, 21, 22, 50].

In addition, *I. simplex* collected in South Africa was morphologically similar to and phylogenetically closely related to European specimens of the same species and therefore did not represent the subspecies *I. simplex africanus* [8]. To our knowledge, this study provided the first sequence available for *I. alluaudi*, a species considered as belonging to the subgenus *Exopalpiger* [3]. This taxonomic position was confirmed by the 16S rRNA phylogenetic analysis here, thus being the only species of this subgenus in the Afrotropical zoogeographic region. This subgenus includes five species occurring in Australasia (*Ixodes antechini, I. fecialis* and *I. vestitus* in Australia; *I. priscicollaris* and *I. goliath* in New Guinea), two species in the Neotropical (*I. andinus* and *I. jonesae*) and two species in the Palearctic (*I. ghilarovi* and formerly *I. trianguliceps*) [51, 52]. Interestingly, *I. alluaudi* from South Africa is located within different Australian *Ixodes* belonging to *Endopalpiger*, *Coxixodes*, *Sternalixodes*, *Exopalpiger*, the newly erected *Australixodes* [53] and *Ceratixodes* and therefore becomes the first molecularly analyzed species in this phylogenetic clade with a geographical range exclusively outside Australia.

*Ixodes uriae* is known to have a worldwide distribution being associated with marine birds [47]. As also shown here, phylogenetic distances may increase with larger scale geographical distance of its samples, but this also depends on host species [54].

In summary, all phylogenetic analyses confirmed the close relationship of subgenera *Afrixodes* and bird-associated *Trichotoixodes*, as suggested based only on mitochondrial genes and a single species from these subgenera [5, 53]. The subgenus *Afrixodes* is geographically bound to Africa and is probably the most species-rich group of the genus *Ixodes*. Moreover, as shown above, the subgenus *Trichotoixodes* also has the highest number of species in Africa. Given the strong phylogenetic support shown here for the common ancestry and short evolutionary distance between subgenera *Afrixodes* and bird-infesting *Trichotoixodes*, we hypothesize that the latter probably also originated in Africa where diversification of *Afrixodes* may have triggered host-switching events towards birds. Considering avian hosts in this context, the African origin of the Passerida songbird radiation, probably following dispersal events from Australia, has been supported by multiple phylogenetic evidence [55, 56]. Consequently, together with their avian hosts, tick species in *Trichotoixodes* may have colonized other continents, except the aboriginal Australia from where they are still absent.

### Supplementary Information

**Additional file 1: Table S1.** Accession numbers of sequences used for the concatenated phylogenetic tree. Sequences from this study are highlighted in bold.

**Additional file 2: Figure S1.** Phylogenetic tree based on the 16S rRNA gene, focusing on Old World *Ixodes *species. In each row of individual sequences, the country of origin and the GenBank accession number are shown after the species name. Sequences from this study are indicated with red fonts and bold, maroon accession numbers. *Ixodes pilosus *group sp. I. and *I. *cf. *rubicundus *are represented by multiple sequences (Table 1), and their branches are shown collapsed. *Rhipicephalus sanguineus *was used as outgroup. The evolutionary history was inferred by using the maximum likelihood method based on the Jukes-Cantor model. The tree is drawn to scale, with branch lengths measured in the number of substitutions per site. The analysis involved 99 nucleotide sequences. All positions containing gaps and missing data were eliminated. There were a total of 304 positions in the final dataset. Evolutionary analyses were conducted in MEGA7.

## Data Availability

The sequences obtained during this study are deposited in GenBank under the following accession numbers. *Cox*1 gene: OQ921940-OQ921984, 16S rRNA gene: OQ924680-OQ924707, 18S rRNA gene: OQ924736-OQ924748, 28S rRNA gene: OQ924930-OQ924948. All other relevant data are included in the manuscript and the references or are available upon request by the corresponding author.

## Abbreviation

cox1: Cytochrome *c* oxidase subunit I
