# Supplementary material for: Molecular-phylogenetic analyses of Ixodes species from South Africa suggest an African origin of bird-associated exophilic ticks (subgenus Trichotoixodes)
Source: Parasit Vectors. 2023 Oct 28;16:392. doi: 10.1186/s13071-023-05998-5 (PMC10612238; doi:10.1186/s13071-023-05998-5)
Supplement: Supplementary file 1 — Additional file 1: Table S1. Accession numbers of sequences used for the concatenated phylogenetic tree. Sequences from this study are highlighted in bold. [file 13071_2023_5998_MOESM1_ESM.docx]

**Additional Table 1.** Accession numbers of sequences used for the concatenated phylogenetic tree. Sequences from this study are highlighted in bold.

| **Species** | **Cox1 gene** | **16S rRNA gene** | **18S rRNA gene** | **28S rRNA gene** |
| --- | --- | --- | --- | --- |
| *Ixodes pilosus* sp. I. | **OQ921940** | **OQ924680** | **OQ924736** | **OQ924930** |
| *Ixodes pilosus* sp. II. | **OQ921974** | **OQ924721** | **OQ924746** | **OQ924946** |
| *Ixodes* cf. *rubicundus* | **OQ921949** | **OQ924690** | **OQ924737** | **OQ924931** |
| *Ixodes* cf. *rubicundus* | **OQ921963** | **OQ924707** | **OQ924742** | **OQ924936** |
| *Ixodes rhabdomysae* | **OQ921961** | **OQ924703** | **OQ924740** | **OQ924932** |
| *Ixodes uriae* (this study) | **OQ921966** | **OQ924710** | **OQ924744** | **OQ924938** |
| *Ixodes uriae* | NC_006078 | NC_006078 | AF115369 | AF120296 |
| *Ixodes ugandanus* | OQ921980 | OQ924730 | OQ924748 | OQ924948 |
| *Ixodes canisuga* | MT659130 | KY962053 | OP998038 | OP998056 |
| *Ixodes hexagonus* | MT659132 | MT658759 | OP998040 | OP998058 |
| *Ixodes kaiseri* | KY962011 | MT658758 | OP998039 | OP998057 |
| *Ixodes lividus* | KU170510 | KU170520 | OP998041 | OP998060 |
| *Ixodes simplex* | KY457531 | KY457531 | KY457498 | KY457498 |
| *Ixodes ariadnae* | KJ490306 | KM455968 | OP998036 | OP998054 |
| *Ixodes vespertilionis* | KJ490307 | KM455966 | OP998035 | OP998053 |
| *Ixodes frontalis* | OL339470 | OL352908 | OP998043 | OP998062 |
| *Ixodes ricinus* | **OQ921983** | **OQ924734** | OP998034 | OP998052 |
| *Ixodes acuminatus* | OL339474 | OL352912 | OP998044 | OP998063 |
| *Ixodes trianguliceps* | **OQ921984** | **OQ924735** | OP998042 | OP998061 |
| *Ixodes holocyclus* | NC_005293 | NC_005293 | AF018650 | AF120294 |
| *Ixodes tasmani* | MN106731 | U95906 | AF115368 | AF120295 |
| *Hyalomma rufipes* | MW884229 | MW884229 | L76349 | KY457486 |
| *Carios vespertilionis* | KX431953 | KX831484 | MT739410 | MT739330 |
