# Supplementary figures and images for: Molecular-phylogenetic analyses of Ixodes species from South Africa suggest an African origin of bird-associated exophilic ticks (subgenus Trichotoixodes)
Source: Parasit Vectors. 2023 Oct 28;16:392. doi: 10.1186/s13071-023-05998-5 (PMC10612238; doi:10.1186/s13071-023-05998-5)

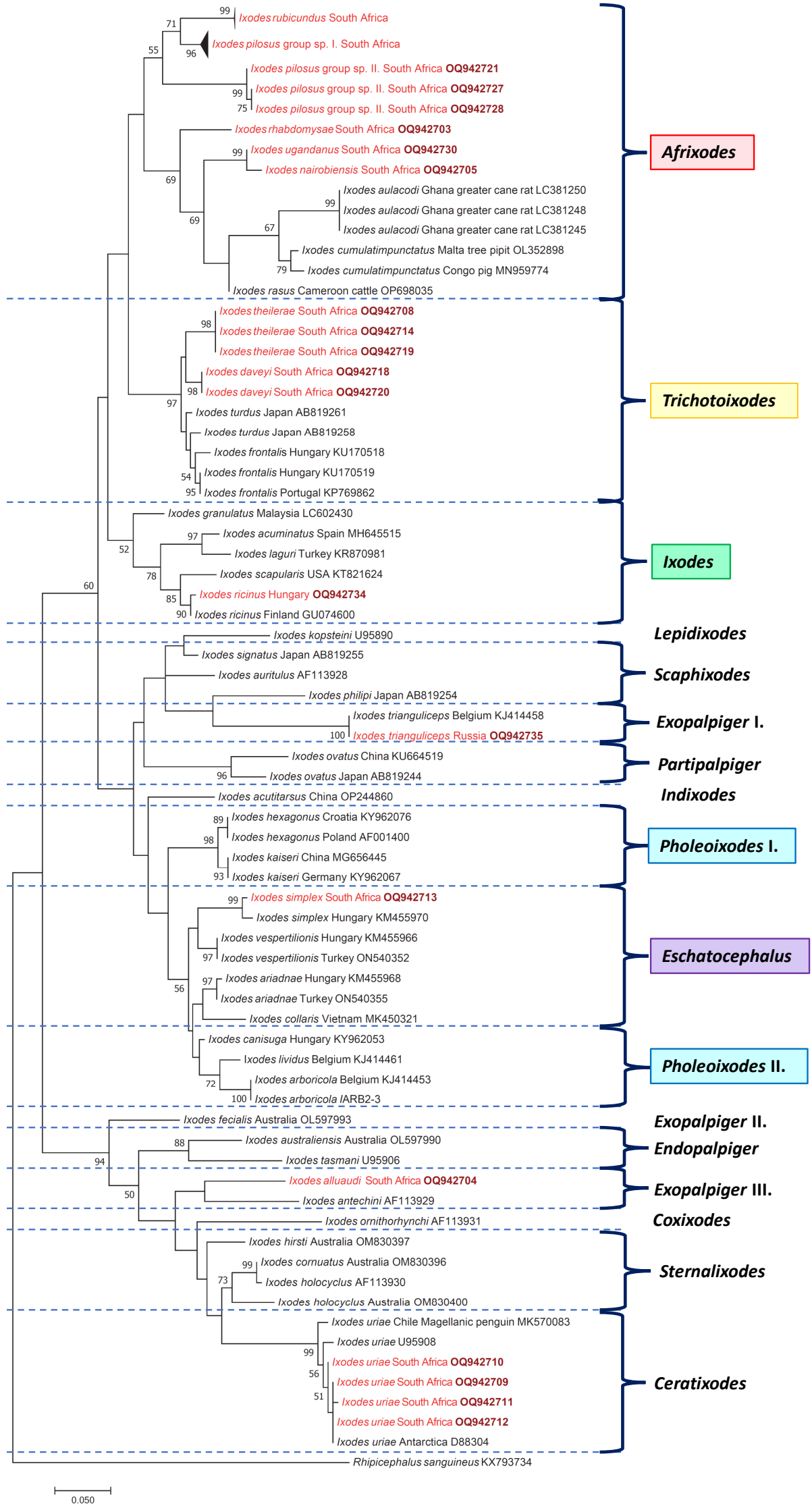

Supplement: Supplementary file 2 — Additional file 2: Figure S1. Phylogenetic tree based on the 16S rRNA gene, focusing on Old World Ixodes species. In each row of individual sequences, the country of origin and the GenBank accession number are shown after the species name. Sequences from this study are indicated with red fonts and bold, maroon accession numbers. Ixodes pilosus group sp. I. and I. cf. rubicundus are represented by multiple sequences (Table 1), and their branches are shown collapsed. Rhipicephalus sanguineus was used as outgroup. The evolutionary history was inferred by using the maximum likelihood method based on the Jukes-Cantor model. The tree is drawn to scale, with branch lengths measured in the number of substitutions per site. The analysis involved 99 nucleotide sequences. All positions containing gaps and missing data were eliminated. There were a total of 304 positions in the final dataset. Evolutionary analyses were conducted in MEGA7. [file 13071_2023_5998_MOESM2_ESM.pdf]
